# Amyloid Beta in Aging and Alzheimer’s Disease

**DOI:** 10.3390/ijms232112924

**Published:** 2022-10-26

**Authors:** Ujala Sehar, Priyanka Rawat, Arubala P. Reddy, Jonathan Kopel, P. Hemachandra Reddy

**Affiliations:** 1 Department of Internal Medicine, Texas Tech University Health Sciences Center, Lubbock, TX 79430, USA; 2 Nutritional Sciences Department, College of Human Sciences, Texas Tech University, Lubbock, TX 79409, USA; 3 Neurology, Departments of School of Medicine, Texas Tech University Health Sciences Center, Lubbock, TX 79430, USA; 4 Public Health Department of Graduate School of Biomedical Sciences, Texas Tech University Health Sciences Center, Lubbock, TX 79430, USA; 5 Department of Speech, Language and Hearing Sciences, School Health Professions, Texas Tech University Health Sciences Center, Lubbock, TX 79430, USA

**Keywords:** Alzheimer’s disease, amyloid beta, amyloid precursor protein, neurofibrillary tangle, mitochondria, therapeutics

## Abstract

Alzheimer’s disease (AD), is a progressive neurodegenerative disease that affects behavior, thinking, learning, and memory in elderly individuals. AD occurs in two forms, early onset familial and late-onset sporadic; genetic mutations in PS1, PS2, and APP genes cause early onset familial AD, and a combination of lifestyle, environment and genetic factors causes the late-onset sporadic form of the disease. However, accelerated disease progression is noticed in patients with familial AD. Disease-causing pathological changes are synaptic damage, and mitochondrial structural and functional changes, in addition to increased production and accumulation of phosphorylated tau (p-tau), and amyloid beta (Aβ) in the affected brain regions in AD patients. Aβ is a peptide derived from amyloid precursor protein (APP) by proteolytic cleavage of beta and gamma secretases. APP is a glycoprotein that plays a significant role in maintaining neuronal homeostasis like signaling, neuronal development, and intracellular transport. Aβ is reported to have both protective and toxic effects in neurons. The purpose of our article is to summarize recent developments of Aβ and its association with synapses, mitochondria, microglia, astrocytes, and its interaction with p-tau. Our article also covers the therapeutic strategies that reduce Aβ toxicities in disease progression and discusses the reasons for the failures of Aβ therapeutics.

## 1. Introduction

Alzheimer’s disease (AD), is a neurodegenerative disease, characterized by memory loss and multiple cognitive impairments. AD is associated with cognitive decline and is the fourth leading cause of death worldwide among the elderly population [1]. AD causes progressive dysfunction and death of neurons, that is why AD patients slowly lose their cognitive abilities and memory [2]. It has been observed that both implicit and explicit memory is affected in AD patients, which means the disease affects a person’s ability to recall recently processed information/events, and also interferes with the phenomenon of improving performance based on earlier experiences or learnings [3]. AD occurs in two forms, early onset familial AD, and late-onset AD, and a greater degree of disturbance in memory is observed in late-onset patients as compared to early onset patients who experience a rapid progression of cognitive decline [4].

**Figure 1:**
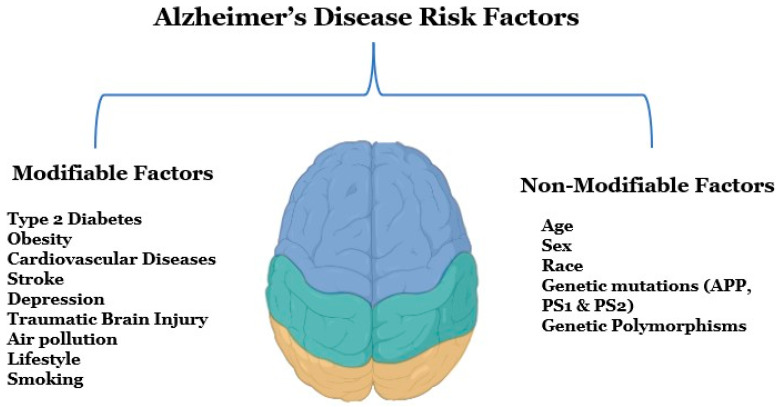
Potential risk factors for Alzheimer’s disease. There are two types of risk factors for Alzheimer’s disease that are modifiable and non-modifiable factors. Modifiable risk factors mainly include diseases, brain injuries, unhealthy lifestyles, and environmental factors, and non-modifiable factors include age, gender, family history, and genetics.

Several modifiable and non-modifiable risk factors are associated with AD (Figure 1). Modifiable risk factors include type 2 diabetes, obesity, vascular diseases, stroke, depression, traumatic brain injury, and various lifestyle factors, whereas non-modifiable risk factors can be genetic morphisms, age, or sex [5]. Early detection and diagnosis are critical for AD, as this disease is a significant public health concern in the United States, according to an estimate, 6.5 million Americans are suffering from AD right now [6]. Alzheimer’s Disease International, an umbrella organization of over 100 Alzheimer’s disease organizations, estimates that around 60 million people globally suffer from dementia, and this figure will reach 78 million by 2030 [7]. Globally, mortality rates from dementia have been increasing rapidly since the 1990s, and the death rate has increased from 10.49 deaths per 100,000 to 20.98 deaths per 100,000 from 1990 to 2019 [8].

**Figure 2:**
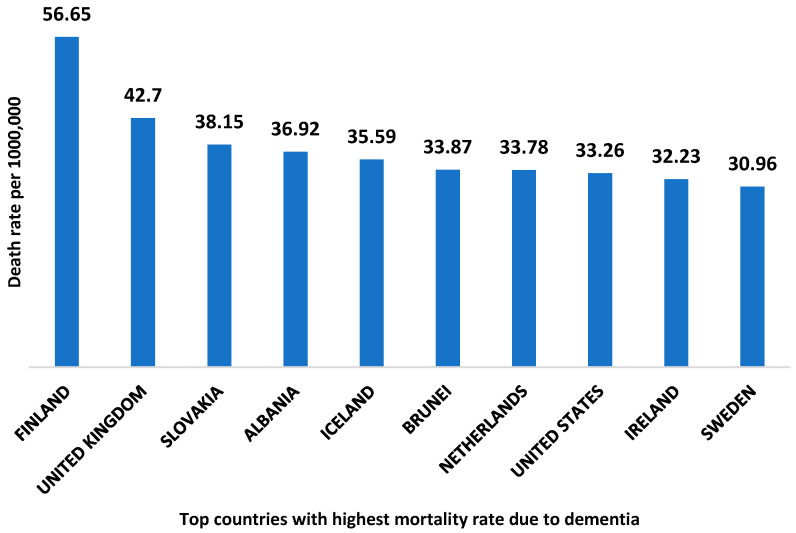
Top ten countries with the highest death rate due to dementia. The data is collected from the World health organization 2020, and the reported death rate is age-standardized.

According to world health organization data, Finland is the country with the highest death rate from dementia with 56.65 deaths per 100,000 [9]. The top ten countries with the highest mortality rate due to dementia are shown in Figure 2. The global economic burden of AD and related dementia was estimated to be $2.8 trillion in 2019. Furthermore, it has been predicted to be increased up to $4.7 trillion by 2030. Moreover, the lower and middle- income countries are expected to account for 65% of the global economic burden of AD in 2050 [10].

Some of the major clinical features of AD are memory loss, cognitive dysfunction, and personality changes [11]. Selective memory impairment is often the earliest clinical manifestation of AD but there is no cure for this disease, only treatments that are available aim to relieve the symptoms [12]. Whereas the pathological causes of the disease include the formation of neurofibrillary tangles (NFTs) made up of an abnormal accumulation of phosphorylated tau protein and the development of senile plaque by amyloid beta in the hippocampus [11]. Aβ is a peptide that is derived from an amyloid precursor protein by proteolytic cleavage. APP is a glycoprotein that plays a significant role in maintaining neu- ronal homeostasis like signaling, neuronal development, and intracellular transport [13]. Whereas Tau protein is a microtubule-associated protein and found abundantly in the neurons of the central nervous system (CNS) primarily involved with the stability of mi- crotubules in axons but is also important for synaptic plasticity, regulation of genomic stability, and cell signaling [14]. Along with the interplay of these proteins, much other health, lifestyles, and environmental factors are culprits causing AD.

The purpose of our article is to provide the most recent and relevant findings on amyloid beta’s structure, function, interactions, and therapeutic strategies.

## 2. Etiology and Pathogenesis of Alzheimer’s Disease

Both genetic and non-genetic factors are believed to be responsible for AD, but a def- inite etiology of the disease is still unclear. Although genetic factors like mutations in APP, PSEN1, and PSE2 genes are known and inherited in the mendelian pattern non-genetic factors like occupational exposures to volatile anesthetics, toxic metals, industrial chemi- cals, electromagnetic fields, air pollutants, and pesticides are environmentally determined. Moreover, some medical pre-existing conditions like diabetes, cerebrovascular disease, hypertension, cancer, depression, traumatic brain injury, and dyslipidemia can also be responsible for the etiopathogenesis of AD (Figure 1). The lifestyle behaviors such as consumption of caffeine, and alcohol, a person’s body mass index, cognitive activity, and physical activities are also important factors [15].

Any long-term exposure to above mentioned environmental contaminants can lead to bioaccumulation of toxins over a person’s lifetime and may induce neuropathology and neuroinflammation that causes the development of AD, however, knowledge of the epi- demiological associations among these contaminants and the prognosis of AD is limited. However, studies on animal models of AD, have shown the lethal effects of environmental contaminants at the cellular level that affects metabolic pathways associated with AD [16]. One of the well-studied factors is the Aβ protein is the principal component of the AD process [17]. Accumulation of Aβ is the initial step of AD and the accumulation starts in the entorhinal cortex and hippocampus of the brain. Moreover, a hyperphosphorylated protein, tau, deposits intracellularly in neurofibrillary tangles changes the cytoskeleton and interrupts axonal transport [18]. The concept of Aβ accumulation being the major event in AD pathogenesis was presented by three independent research groups in 1991 [19,20,21].

## 3. Amyloid Beta Precursor Protein Processing

In AD patients, the digestion of APP is done by cumulative action of alpha- and gamma-secretases that produce insoluble peptides, amyloid-beta, which cluster together to form amyloid beta plaques thus degenerating cells [22]. In healthy brains, cleavage of Aβ is done by beta-secretase enzymes resulting in the formation of soluble APP fragments, and the rest of the APP is further cleaved by γ-secretase-producing peptides that are released outside the cell and rapidly removed/degraded. However, in elderly people, the secretase homeostasis is dysregulated, and APP is cleaved by β and γ-secretase and produces insoluble amyloid beta peptides [23].

**Figure 3:**
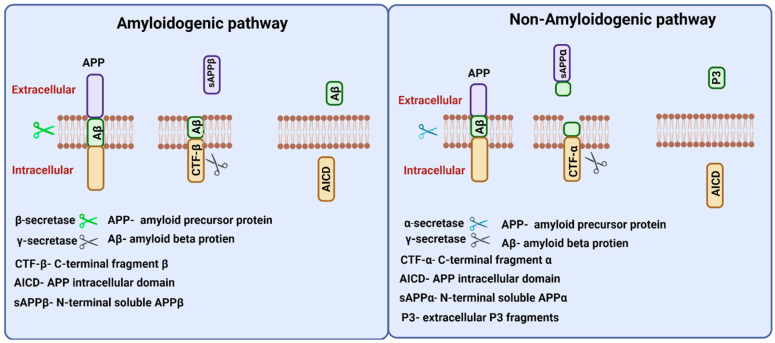
Amyloid precursor protein (APP) processing. Two proteolytic pathways, amyloidogenic and non-amyloidogenic processing, exist for APP processing. The amyloidogenic pathway involves β and γ secretases and releases N-terminal soluble APPβ fragments, and Aβ peptides in the extracellular region. The non-amyloidogenic involves α and γ secretases and releases N-terminal APPα fragments, and P3 peptides in the extracellular region. Both proteolytic pathways release APP intracellular domain (AICD) fragments intracellularly.

APP gene in humans is located on chromosome 21 and alternate splicing of this gene produces 8–11 isoforms of APP protein of different amino acid lengths. APP is a transmembrane protein that is transported through secretory and endocytic pathways [24]. Among its isoforms, APP 751 and APP 770 are found to be expressed in glial cells, and provide support for neurons, and APP 695 is expressed in neurons [25].APP proteolysis in humans follows amyloidogenic and non-amyloidogenic pathways (Figure 3). The amyloidogenic pathway includes the processing of APP by the action of β- and γ-secretases. β-Secretase (BACE-1) cuts APP into the membrane-bound fragment known as C-terminal fragment β (CTFβ) and N-terminal soluble APPβ (sAPPβ) [26]. CTFβ is further cleaved by γ-secretases and generates extracellular Aβ protein fragments and APP intracellular domain (AICD) [27]. The non-amyloidogenic pathways process APP by the action of membrane-bounded enzyme α-secretase that cuts within the Aβ sequence and generates membrane-bound C-terminal fragment CTFα and N-terminal fragment sAPPα. Further processing of CTFα yields extracellular P3 fragments and APP intracellular domain (AICD) [26].

### 3.1. Amyloid Beta

Amyloid beta (Aβ) is the cleavage product of a glycoprotein, amyloid precursor protein (APP), and is normally present in the brain as part of the signal transduction process [28]. The dysregulation of Aβ levels in the brain leads to the formation of senile plaque, and deposition of Aβ, which causes cognitional disabilities in patients with AD [29]. APP is a transmembrane protein that has three domains: one inside the cell, one in the cell membrane, and one protruding out of the cell. The domains of APP are digested by alpha-, beta- and gamma-secretases. APP cleavage by β-secretase followed by γ-secretase cleavage yields various lengths of Aβ peptides like Aβ_42_ and Aβ_40_ [30]. These two major isoforms of Aβ differ in the number of residues as Aβ_42_ has two extra residues at its C-terminus as compared to Aβ_40._ The Aβ isoform that forms amyloid plaques in AD is mostly Aβ42, and some plaques only contain the Aβ42 isoform [31].

The insoluble Aβ accumulates in AD because of an imbalance between the production and removal of these peptides from the specific parts of the brain. The majority of AD is sporadically caused by inefficient removal of Aβ peptide, and less common AD is caused by mutations in genes linked to Aβ metabolism and is known as familial AD [32]. Sporadic AD is usually known as late-onset, whereas, familial is known as early onset and is inherited usually in a mendelian fashion [33]. Familial AD is a very rare, early onset, an autosomal-dominant disease caused by mutations in the APP and the presenilin gene, both of which are involved in Aβ metabolism. Unlike familial AD, sporadic Alzheimer’s is very common with more than 15 million people affected worldwide [34].

The etiology of sporadic AD is unknown, but possible reasons can be heterogeneity, caused by aging along with the complex interplay of genetic and environmental risk factors. In treating AD, a possible therapeutic strategy is reducing Aβ levels in the brain which can be achieved by inhibiting the generation of Aβ by targeting APP or secretases, triggering mechanisms that clear Aβ species, or developing mechanisms that balance production and clearance of Aβ peptide in the brain [35].

#### 3.1.1. Functions of Amyloid Beta

**Figure 4:**
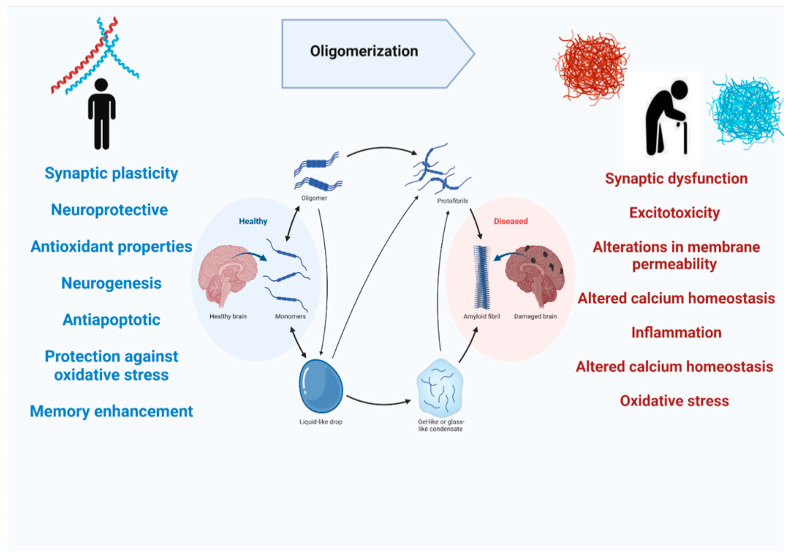
The physiological and pathological role of amyloid beta in the human brain.

Most of the studies on Aβ are mainly focused on its pathological role in causing cognitive decline and resulting in Alzheimer’s disease. On the contrary, APP does have normal physiological functions in the brain. APP knockout mice have been found to have smaller brains and alterations in neurogenesis have been observed [36]. Moreover, APP has its role in promoting neurite outgrowth in the nervous system, and it is also involved in long-term potentiation, strengthening synapses to increase signal transmission, by regulating calcium release (Figure 4) [37,38]. Aβ peptide is notoriously known for their deposition in the brain during the disease process, but they are also released in normal brains throughout our lives during synaptic activity [30]. The in vivo studies on rodents showed that picomolar concentrations of Aβ in the brain can regulate synaptic plasticity. Studies also showed that Aβ _40_ in picomolar levels can play a neurotrophic role in cell cultures [39,40], also Aβ_42_ is found to increase the number of neurons in neural stem cell cultures [41]. Aβ peptide has also been shown to possess neuroprotective and antioxidant properties. It has been demonstrated that Aβ can reduce apoptotic death in neuronal cultures when administrated in nanomolar concentrations [42], and this antiapoptotic behavior is linked with Aβ’s chelating ability [43]. The amyloidogenic pathway of APP processing yields soluble, secreted protein fragments along with membrane-associated fragments [44]. The soluble Aβ fragments play physiological roles like synaptic plasticity, neurogenesis, and long-term potentiation but as this soluble Aβ binds to other Aβ and forms oligomers that cleared from the brain more slowly and they aggregate to form insoluble Aß plaques. The oligomeric and insoluble Aβ is neurotoxic as they aggregate to form plaques and causes AD [45]. The toxic mechanisms of Aβ may include synaptic dysfunction, excitotoxicity, alterations in membrane permeability, altered calcium homeostasis, inflammation, altered calcium homeostasis, and oxidative stress [43].

In 2005, a study by Grim and colleagues [46] reported that Aβ is involved in the regulation of cholesterol and sphingomyelin metabolism. There are two main enzymes, sphingomyelinases, and Hydroxymethylglutaryl-CoA reductase, that regulates cholesterol biosynthesis, and it has been observed that Aβ42 activates sphingomyelinase that downregulates sphingomyelin biosynthesis. Furthermore, Aβ_40_ inhibits Hydroxymethylglutaryl-CoA reductase activity and reduces cholesterol biosynthesis [46].

Several studies showed the complete absence of APP genes in transgenic mice causes severe neurological deficits [47] and the neuron cell culture that were subjected to β- or γ-secretase inhibitors ended up with neuronal cell death [40]. Growing data on APP and Aβ suggests that they play important physiological functions in the brain and Aβ is only toxic when the balance between its production and clearance is disrupted.

#### 3.1.2. Amyloid Beta and Beta-Sheet Formation

The soluble and low molecular weight Aβ oligomers are aggregated in the early phases of disease progression and are believed to be a major toxic entity of amyloidogenesis, the formation/growth of amyloid structure in neurodegenerative diseases [48]. The oligomers, amyloid fibrils, and protofibrils formation from the Aβ peptide is the hallmark of AD. Such structural assemblies derived from Aβ are very crucial for the onset of AD, and determining their atomic structures has been a major challenge [49]. Structural studies of Aβ-derived assemblies have revealed meaningful details about such assemblies at different structural levels. The studies have shown that Aβ structural conformation changes from α-helix to a β-sheet structure and amyloid fibril assembly is initiated by a mechanism that is nucleation dependent and leads to the elongation of these fibrils. During this mechanism protofibrils, short fibrillar structures, and intermediate small oligomeric structures are also seen. The cross-sectional study of fibrils revealed that they are composed of several subfibrils or protofilaments, and the protofilaments are themselves composed of a β-sheet structure where hydrogen bonding occurs along the length of the fibre and β-strands found running perpendicular to the fibre axis [50]. The occurrence of β-sheet structures encourages Aβ’s toxic roles in the progression of AD and makes it a therapeutic target [48].

#### 3.1.3. Amyloid Beta in Familial AD

Familial AD exhibits typical neuropathological hallmarks of sporadic AD-like tissue atrophy and neuronal loss, neurofibrillary tangles, and amyloid plaques in enhanced higher amounts [51]. Familial AD is caused by missense mutations in APP, presenilin-1 (PS1), and presenilin-2 (PS2) [52]. The first genetic factor causing AD reported was the occurrence of missense mutations in APP [53]. The mutations in APP are located after the α-secretase site, before the β-secretase cleavage site, after the α-secretase site, or COOH-terminal to the γ-secretase cleavage site. Despite the research, no other mutations in APP were found to cause AD proving that these missense mutations alter the proteolytic processing of APP by three secretases and lead to AD [52]. Although many mechanisms lead to AD progression, the amyloid cascade is still one of the most studied theories linked to AD, and the association of mutations in APP/PSI/PS2 with amyloid synthesis and processing further strengthens the role of amyloid beta in familial AD than the sporadic one [54].

#### 3.1.4. Amyloid Beta in Sporadic AD

Sporadic AD, non-mendelian AD, is the most commonly occurring type of dementia worldwide [55]. It is a multifactorial disease caused by diverse genetic factors that have been identified by large-scale genome-wide studies, and more than fifty genes are suspected to be associated with sporadic AD risk [56]. Among others aging itself is a risk factor to develop sporadic AD. Intracellular Aβ peptides initiate a series of pathological events extending to synaptic dysfunction, mitochondrial dysfunction, loss of calcium regulation to inflammation, and oxidative stress, the combined effect of these events causes progressive loss of neurons. The formation of Aβ hinders mitochondrial function which is an early event in the progression of AD. On the contrary, mitochondrial impairment particularly increased the formation of mitochondrially derived reactive oxygen species, that activate beta and gamma secretases and promote Aβ formation [57]. The large-scale genome-wide studies showed that genes linked to Aβ regulation include the ones involved in its expression, transmission, and degradation. Furthermore, some genes linked to the ubiquitin-proteasome pathway, and endosomal-lysosomal system are associated with Aβ homeostasis. The list of genes involved in Aβ homeostasis in sporadic AD is listed in Table 1 [58].

### 3.2. Amyloid Beta and Synapse

Although accumulation of Aβ is one of the hallmarks of Alzheimer’s disease, synaptic loss and dysfunction are also linked to the severity of cognitive deficit in AD patients [59]. Aggregation of Aβ occurs as the result of either an increase in Aβ production or a decrease in its clearance and these aggregates vary from insoluble senile plaques to soluble Aβ monomers and oligomers [60]. AD brains are also characterized by severe synaptic loss in mesial temporal regions that includes the hippocampus, parahippocampus, and amygdala [61]. The studies have shown that the immediate vicinity of Aβ plaque is the prominent area where synaptic loss has been observed and may be the reason that Aβ serves as a synapto-toxic molecule [62]. The dendritic spine, a small membranous protruding structure found on a neuron’s dendrite, is enriched with a cytoskeleton protein known as helical filamentous actin (F-Actin). Kommaddi and colleagues demonstrated that amyloid beta-mediated disassembly of F-actin and dendritic spines lead to cognitive decline in AD patients as either an increase in G-actin protein or a decrease in F-actin can lead to the onset of AD or the disease itself is a consequence of dendritic loss [63]. An increase in Aβ production has been reported with the progression of AD, and this accumulation of Aβ adversely affects synaptic plasticity and also hinders synaptic vesicle trafficking [64].

A recent study conducted by Torres-Flores, and Pena-Ortega (2022) has revealed that Aβ disturbs synaptic plasticity in male rats and affects prefrontal cortex-dependent functions that include working memory and cognitive flexibility. The study evaluated the effects of Aβ injection into the prefrontal cortex, and it has been observed that a single Aβ injection learning and performance of animals in delayed nonmatching to sample test that is used to check working memory in animals [65].

### 3.3. Amyloid Beta and P-Tau at Synapses

The amyloid cascade hypothesis suggests Aβ deposition occurs first and then triggers tau pathology [66], but the neuropathological connection among these is not yet very well defined. Furthermore, Aβ accumulation at synapse precedes phosphorylated tau (p-tau) [64]. Fein and colleagues [66] studied the co-localization and regional distribution of Aβ and p-tau in synaptic terminals of AD brains. They found Aβ and tau pathology overlapping consistent with a model where synaptic loss and synaptic dysfunction were linked to a synaptic amyloid cascade in the synaptic compartment [66].

**Figure 5:**
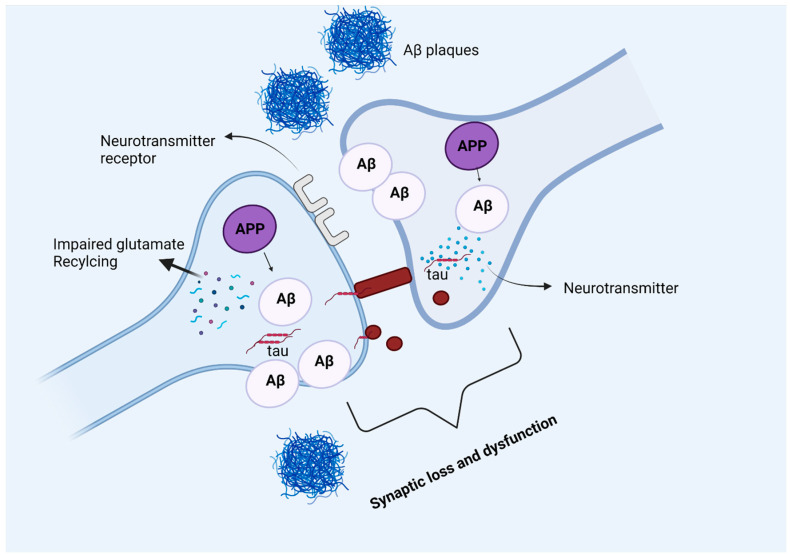
Amyloid beta and tau protein at the synapse. In Alzheimer’s disease, amyloid precursor protein is processed into amyloid beta peptides that accumulate inside and outside the neuronal cells and form plaques. The deposition of amyloid plaques in the vicinity of the synapse causes synaptic loss and dysfunction. Moreover, glutamate recycling is also dysregulated in the presence of pathological amyloid beta levels in neurons. The soluble, hyperphosphorylated tau protein can directly move across the plasma membrane, or by another mechanism where the formation of nanotubules helps the translocation of tau intracellularly. The synergetic pathology of Aβ and tau at synapse causes synaptic loss and dysfunction.

More recently Bilousova and colleagues conducted a study to determine the sequence by which Aβ and p-tau pathologies appear in synapses. For this, they quantified Aβ and p-tau across AD disease developmental stages in the parietal cortex of human subjects and rodent models [67]. Their study suggests that Aβ drives p-tau accumulation as p-tau increases with Aβ fractions at synapses. Thus, it can be assumed that p-tau induction is driven by synaptic oligomeric Aβ, refer to Figure 5. Such findings support the fact that anti-amyloid therapeutics will be less effective once p-tau pathology has been developed.

### 3.4. Amyloid Beta and Mitochondria

Along with the major features of AD, Aβ plaques, and neurofibrillary tangles, the disease is also associated with inflammatory responses, loss of neurons, synapses, and mitochondrial abnormalities and these features have been reported as early events in disease progression [68]. Amyloid beta affects the morphology and biochemistry of mitochondria, and this interaction interferes with mitochondrial functions like reactive oxygen species (ROS) production, energy metabolism, and pore formation. Studies have shown that Aβ accumulates in the mitochondrial matrix over time and causes mitochondrial toxicity [69]. APP and amyloid beta is known to localize to membranes of mitochondria thus blocking the transport of mitochondrial proteins encoded by the nucleus to mitochondria and interrupting the electron transport chain, damaging mitochondria, increasing ROS production, and disrupting neurons functionality [70]. Moreover, amyloid beta-induced mitochondrial dysfunction leads to mitochondrial DNA damage, phosphorylation of tau, Aβ-Drp1and P-tau-Drp1 interactions, binding of Aβ with alcohol dehydrogenase, and loss of cardiolipins and cytochrome c oxidase activity [71].

### 3.5. Amyloid Beta Interaction with Brain Proteins

Amyloid beta and tau proteins are principal components in the development of AD. Tau protein, a microtubule-associated protein, normally performs the function of stabilizing microtubules A lot of research has been done on both the proteins and their separate modes of action in the progression of AD. Recent studies have accumulated evidence on the interaction between these proteins and their synergistic mode of action in the pathogenesis of AD [72]. The advances in mouse models, image analyses, and biomarker studies have redefined the original amyloid beta hypothesis and it is amyloid beta and tau proteins along with other mechanisms like inflammation lead to the development of AD [73].

The current AD models advocate the role of Aβ, either in soluble, plaque, or non-fibrillar form, in tau misfolding and assembly that in turn expands into the cortex and eventually disrupts the neural system and results in cognitive decline [72]. In later stages of AD, cortical decay and synaptic loss have been an indication of AD progression due to amyloid beta and tangle’s structural damage to the brain, whereas memory loss in the earlier stages of AD has been reported by the combined effect of soluble Aβ and tau at the synapse [74]. Furthermore, several studies showed that administrating various preparations of tau and oligomeric Aβ results in memory loss and impaired synaptic plasticity [75].

### 3.6. Amyloid Beta and Cellular Changes

**Figure 6:**
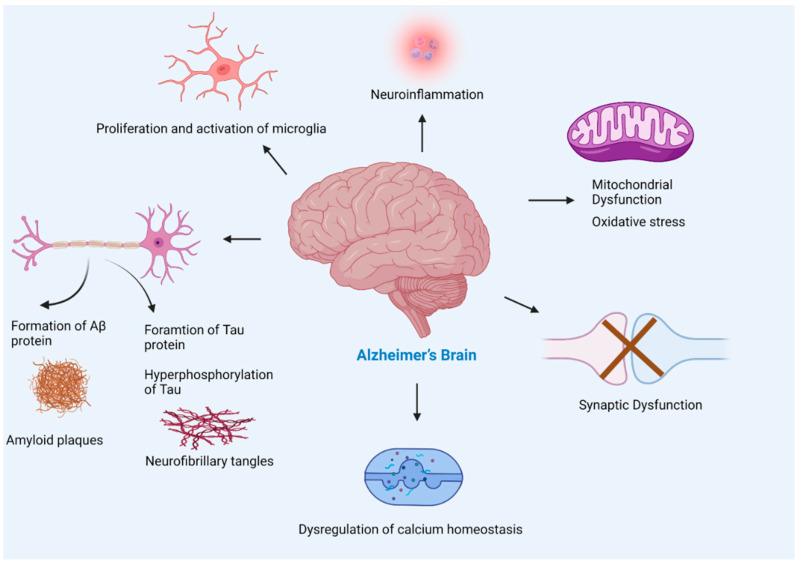
Pathogenesis of Alzheimer’s disease. The multiple factors responsible for the progression of Alzheimer’s disease include amyloid beta plaques, and tau neurofibrillary tangles formation leading to neuronal loss, activation of microglia (found to be concentrated in the vicinity of amyloid plaques) that leads to neuroinflammation, mitochondrial dysfunction, synaptic loss, and dysregulation of calcium homeostasis. The accumulation of Aβ in Alzheimer’s brain disturbs synapsis which leads to postsynaptic hyperexcitability. The hyperexcitability of neurons causes dysregulation of calcium homeostasis and an increase in the production of reactive oxygen species.

AD causes several changes in the brain due to the complex interplay of amyloid beta plaques and abnormal tau that affects brain communication, refer to Figure 6. Aβ leads to impaired cellular respiration, disrupts energy production, and interferes with mitochondrial activities. Moreover, deposition of Aβ causes synaptic damage, neuroinflammation, and oxidative stress. The following section discusses how amyloid beta changes cellular normal physiological functions.

#### 3.6.1. Amyloid Beta and Synaptic Damage or Loss

In the AD brain, loss of the synapse is one of the major connections to cognition decline [76,77]. Many pieces of evidence suggest that soluble oligomer Aβ induces a synaptic loss in AD [78]. Pathological Aβ oligomer interacts with multiple astrocytic, microglial, and neuronal synaptic proteins including α7-AChRs and NMDARs, and provokes multiple toxic synaptic events [78]. Spine loss due to Aβ causes the disintegration of the neuronal network over time as a consequence individuals with AD lose their cognitive ability [77]. In AD, synapse degeneration extensively has been found close to amyloid plaques [79], and this may be caused by increased levels of Aβ oligomers rather than the plaques themselves [80]. Aβ peptides take part in glutamatergic neurotransmission and alter both pre and postsynaptic mechanisms [81]. Aβ oligomer binds to the synaptic receptor including cellular prion protein, NgR1, EphB2, and PirB/LilrB2, and this Aβ oligomer cause calcium influx and synaptic impairment [82]. Mutation in genes encoding a synaptic protein, alteration in intrinsic synaptic molecular mechanisms, and alteration in the biochemical processes in the surrounding environment of synapse cause synaptic impairment [83].

#### 3.6.2. Amyloid Beta and Inflammation

In AD brains, neuroinflammation plays a significant role in neurodegenerative pathways. Especially, Aβ is known to provoke inflammatory responses that result in neurodegeneration due to synaptic dysfunction, and neuronal death [84]. The key players causing neuroinflammation in AD brains are microglial cells, astrocytes of the brain, cytokines, chemokines, pentraxin acute-phase proteins, neuronal-type nicotinic acetylcholine receptors (AChRs), peroxisomal proliferators-activated receptors (PPARs), and alternate pathways of the complement system [85]. An imbalance in the equilibrium of anti-inflammatory and proinflammatory signalling causes neuroinflammation [86].

The studies have shown that innate immunity is initiated by the interaction of various Aβ complexes with microglial and astrocytic expressed pattern recognition receptors. The neuroinflammation process entails the secretion of pro-inflammatory proteins cytokines, and chemokines along with the formation of reactive oxygen species that, in abundance, dysregulates immune response and contributes to neurodegeneration. The mechanisms by which Aβ production, aggregation, and clearance are influenced by neuroinflammatory response are becoming an interesting target area for therapeutic interventions for AD [87].

#### 3.6.3. Amyloid Beta and Activated Microglia

In CNS microglia are the primary immune effector cells and are the major cellular mediator of the neuroinflammatory response in AD [88]. Once the microglial cell is activated, its morphological and biological roles are altered, and it can initiate an immune response [88]. In the AD brain, Aβ is the main reason for the activation of microglial cells, these activated microglial cells respond to the Aβ plaques and phagocytosis of Aβ [86]. Microglia activation caused by Aβ results in the secretion of proinflammatory cytokines including IL-1β, IL-6, and TNF-α [89]. Secretion of these proinflammatory cytokines and linked neurotoxins results in more microglia activation and contribute to neurodegeneration [86].

#### 3.6.4. Amyloid Beta and Astrocytes

The physiological role of astrocytes is to regulate brain functions that are connected to synaptogenesis, and neurogenesis, maintain extracellular homeostasis and control blood–brain barrier permeability [90]. Moreover, when astrocytes interact with neurons they play their role in energy metabolism regulation, synaptic remodeling, modulation of oxidative stress, information processing, signal transmission, neurotransmitter secretion, and recycling, and extracellular ion homeostasis [91,92]. Studies have shown that astrocytes play important role in the progression of AD by secreting Aβ in significant amounts and contributing to the accumulative amyloid burden in the AD brain. Since astrocytes are abundantly found cell types in the brain, a minor contribution of Aβ secretion by them could result in a substantial increase in the Aβ deposits across the brain. Reactive astrocyte species possess increased levels of APP, secretase (BACE1) and γ-secretase that are components for Aβ production [93].

#### 3.6.5. Amyloid Beta and Hormones

According to many studies, reproductive hormones such as estrogen, progesterone, testosterone, and luteinizing hormone also act as neuroprotective, and any alteration in the level of these hormones is known to increase the risk of AD [94]. A marked increase in luteinizing hormone, a reproductive regulator, followed by menopause/andropause has been reported as a physiologically important signal that can Aβ deposition in the aging brain [95]. A more recent study has reported the Aβ degeneration and clearance pathways at choroid plexus epithelial cells may compromise due to age, sex hormones, and circadian disturbances [96]. The choroid plexus epithelial cells serve as an important route for Aβ clearance. This route either facilitates Aβ transport from the cerebrospinal fluid to the blood or secretes important proteins that are involved in Aβ degradation and clearance. Any disturbance of choroid plexus synthesis may lead to the disruption of Aβ homeostasis in the brain [96]. Similarly, Estrogen also affects Aβ homeostasis by increasing Aβ protein uptake by microglia cells derived from the human cortex [97], and estrogen is also known to play the role of neuroprotection by preventing neurons from Aβ-induced apoptosis [98]. Some studies suggest that any alteration in estrogen level during aging increase the risk of AD [99]. The loss of sex steroid hormones estrogen 17β-estradiol in women and testosterone in men, in normal aging, has increased the risk of AD due to the loss of neuroprotective action of the hormones as the brain is a hormone-responsive organ [100].

#### 3.6.6. Amyloid Beta and Oxidative Stress

The process of oxidative stress increase in the brain with aging, and is caused by an imbalance in the redox state, excess production of ROS (reactive oxygen species), and dysfunction in the antioxidant system [101,102]. The brain is mostly composed of easily oxidized lipids and has a high consumption rate of oxygen and a lack of a strong antioxidant defence system [102]. Electron transport of aerobic respiration in the mitochondria generates ROS as a by-product, and an excess amount of these ROS leads to oxidative stress [103]. Oxidative stress seriously damages the brain through an interacting process including an increased concentration of intracellular free Ca^2+^, release of excitatory amino acids, and neurotoxicity [101].

Since Aβ plaques are a hallmark of AD, they are formed by the aggregation of Aβ with metal ions such as zinc, copper, or iron. Redox metal ions upon binding with Aβ catalyze the production of ROS, thus the ROS produced is the most reactive one and is hydroxyl radical, leading to oxidative damage to Aβ and surrounding molecules like protein, and lipids [104]. At the molecular level source of oxidative stress are lipid peroxidation, protein oxidation, DNA oxidation and glycoxidation, and the production of toxic species, such as peroxides, alcohols, aldehydes, free carbonyls, ketones, cholestenone, and oxidative modifications in nuclear and mitochondrial DNA [105].

#### 3.6.7. Amyloid Beta and Mitochondrial Abnormalities

A growing body of shreds of evidence suggests that is the common pathological mechanism in AD due to the formation of Aβ plaques and neurofibrillary tangles, oxidative stress, neuroinflammation, impairment in the cholinergic system, and dysfunction in synaptic transmission and plasticity [106]. Energy metabolism dysfunction is one of the earlier and more consistent symptoms of AD [103]. Increased concentration of ROS leads to molecular damage at the site where they generate and the surrounding areas by the process of diffusion [107]. The enzymes involved in mitochondrial energy production such as complex IV cytochrome c oxidase, pyruvate dehydrogenase complex, mitochondrial isocitrate dehydrogenase, α-ketoglutarate dehydrogenase, and ATP synthase complex are found to decrease, while the succinate dehydrogenase (complex II) and malate dehydrogenase activities are increased in AD brains and these results in compromising inner membrane potential and the mitochondrial ATP production [107].

## 4. Conditions That Impact Amyloid Beta Toxicity

Emerging evidence suggests that conventional cardiometabolic risk factors including insulin resistance, hypertension, central obesity, inactive lifestyle, diabetes, and cardiovascular disease are linked with the advancement of cognitive decline and AD [108]. A healthy lifestyle can protect against AD and associated diseases, including diabetes and vascular disease [109]. The role of lifestyle factors such as diet and exercise can directly modify the chance of developing the disease [110].

Diet. Nutrition is one of the key modifiable risk factors which can play a role in preventing or delaying the onset of dementia [111]. According to many studies on health behaviour, it has been suggested that consuming fruits and vegetables helps in maintaining an optimal level of blood pressure, blood cholesterol, and weight that affects AD directly or indirectly by preventing associated diseases [109]. Polyunsaturated fatty acids, polyphenols, and antioxidants are neuroprotective, improve brain health, and reduce the risk of AD [110]. Healthy nutritional food is rich in antioxidants and exhibits anti-inflammatory properties, which are responsible for regulating the immune system and can alter the neuroinflammatory events involved in the progression of cognitive decline and AD [108].

Many in vitro and in vivo studies showed that vitamin C decreases Aβ oligomer formation and oxidative stress [112]. Some foods such as meat, and high-dairy products enriched with saturated fat enhance cognitive ability [113]. A ketogenic diet is also found to be effective against neurodegenerative disease [113].

Physical exercise. Physical inactivity is a modifiable risk factor for a variety of diseases including cardiovascular disease to other chronic diseases such as diabetes mellitus, obesity, hypertension, depression, osteoporosis, osteoarthritis, colon cancer, and breast cancer [114]. Regular physical exercise is an integral part of a healthy lifestyle. Daily physical activity reduces the risk of cognitive decline including dementia [115]. Moreover, physical activity directly affects the body’s organs like the brain and heart. Physical exercise affects dopaminergic, serotonergic, and noradrenergic pathways [116].

Sleep. Sleep is one of the important factors that play a significant role in the pathogenies in AD. Sleep plays a restorative function in the brain and aids in memory retention [117]. Good quality sleep is a day and a night cycle, where activity in the day is followed by sleep at night. Several studies have found that sleep changes cellular structure, and thus affects the Aβ clearance mechanism of the brain. A team of researchers from the University of California conducted a study where they measured the Aβ burden in the brain of 20 healthy individuals after a night of full sleep and after a night of sleep deprivation [118]. It has been observed that one night of sleep deprivation resulted in a significant increase in Aβ burden in the thalamus and right hippocampus of the brain as compared to the Aβ levels after a normal night of full sleep. Lucey and colleagues [117] proposed having an adequate sleep of 8–9 h to mitigate the risks of the development of AD in the future.

Brain/Mental Health. The capacity of the brain to adapt to transitional conditions is called brain plasticity, and it depends on the ability of neurons to alter the composition and strengthen their connection in response to external as well as internal stimuli [119]. We can delay or reduce the risk of age-related cognitive decline and neurodegeneration through exercise [120]. Much evidence is considered that physical exercise encourages structural and functional changes in the brain by acting as a strong gene modulator [121]. Aerobic and external resistance exercise helps muscles in releasing/producing brain-derived neurotrophic factors and metabolites in the blood, and these molecules pass through the blood–brain barrier to alter the effect of neuron and glial cells of the brain [119]. Exercise can enhance the regulation of neuronal cell proliferation and accelerate their ability to maintain neuronal plasticity [120]. Regular exercise for three months increased the volume of the blood in the dentate gyrus as evaluated by functional magnetic resonance and improve the cognitive score in humans [120]. Depending on earlier research, it has been established that exercise increases cerebral blood volume, improves memory, and supports perfusion of the hippocampus, a 12 week of physical training regime would enhance cerebral blood flow in the hippocampus, and will improve cognition (memory and executive functions) [122].

There is so much emerging literature that reports the positive effect of exercise on mood including anxiety stress, and depression by physiological and biochemical processes [123]. Exercise also reduces inflammation by different mechanisms that include the participation of cytokines, Toll-like receptors, and adipose tissue and results in overall good health in people suffering from mood disorders [123]. Much evidence suggests that physical activity and exercise improves social skill, self-image, and self-confidence and reduces anxiety, depression, and mental/psychological effects [124].

Regular exercise is directly involved in alleviating cardiovascular mortality as well as reducing the risk of cardiovascular disease as cardiac and vascular changes have been associated with different changes in tissue metabolism and signalling [115]. Obesity is one of the major reasons for many health issues as it enhances the risk of cardiovascular disease, type II diabetes, and certain types of cancer [125]. Physical exercise ameliorates the antioxidative protective mechanism by delaying the accumulation of reactive oxygen species that mediates cell damage in the myocardium [126]. Data from numerous epidemiological studies show that a low level of physical activity is linked with a higher prevalence of most cardiovascular-related conditions such as hypertension, obesity, metabolic syndrome, type II diabetes, and depression [127].

Impact on synaptic function: Exercise has a great impact on the hippocampal region of the brain in the sense that it can bring changes in the neural activity of this area, presumably by boosting learning and memory via short- and long-term changes in synaptic plasticity [128]. An increasing number of studies espouse the thought that brain functioning is increased throughout life due to physical exercise [129]. After exercise, the structural changes in the neurons such as spine density, dendritic complexity, and maturation of new-born neurons in the adult hippocampus, are highly linked with functional changes [129].

Type 2 Diabetes. Type 2 diabetes comes under the metabolic disorder category characterized by insulin resistance, glucose metabolism dysfunction, and hyperinsulinemia and ultimately leads to β cell destruction in the pancreas [130]. According to growing pieces of evidence, type 2 diabetes are linked with dementia and neurodegenerative disease such as AD [131]. Brain atrophy decreased cerebral glucose metabolism, and CNS insulin resistance is a feature of both AD and type 2 diabetes [131]. Both diseases exhibit common features such as cognitive decline, and inflammation [132]. Inflammation caused by Aβ further enhances the production of Aβ 42 peptides [132]. Neuronal insulin resistance cause Aβ accumulation, because the insulin-degrading enzyme also degrades Aβ [132]. Insulin degrading enzyme is not able to degrade and clear Aβ oligomer accumulation that acts as a competitive substrate for insulin, and this insulin resistance facilitates the production of both Aβ and tau oligomer [133].

Down Syndrome. Individuals with Down syndrome are at a lifetime risk of dementia above 90% [134]. Down syndrome individual has trisomy on chromosome 21, and an extra copy of APP causes overproduction of Aβ due to increased APP expression by proteolytic processing [134]. In many individuals with down syndrome it is observed that over the age of 45, the accumulation of plaques and tangles gradually increases [135]. Many studies suggest that accumulation of Aβ can occur in the early stage but in three and four decades of life, Aβ deposition is often observed systematically [136]. In down syndrome, during aging and development, the accumulation of soluble Aβ may compromise cellular function and lead to impairment in specific neurotransmitter systems [135].

Traumatic Brain Injury. Traumatic brain injury is one of the key risk factors for the development of AD, Aβ is seen in 30% of patients who died from TBI [137].TBI initiates alteration in several molecular, biochemical, and cellular processes that cause neuronal damage and cell death [138]. Repetition of this damage provoked multiple multidirectional actions such as oxidative stress, excitotoxicity, apoptosis, inflammation, cerebral metabolism, and mitochondrial dysfunction [138].

### 4.1. Diagnosis of Alzheimer’s Disease

Several methods and tools have been used to determine cognitive deficits that are responsible for AD in patients. Many clinicians used standardized mental status scales to document the presence and progression of dementia [12]. The AD diagnosis is based on tests doctors conducted to evaluate memory and thinking skills. Physical and neurological examination includes muscle tone and strength, sense of sight and hearing, coordination, and balance.

Currently, available diagnosis for AD includes mini-mental state examination (MMSE) evaluations, *cerebrospinal fluid* (CSF) assay for Aβ, magnetic resonance imaging (MRI) for brain volume, and positron emission tomography (PET) scan for Aβ plaques and alterations in glucose metabolism [139].

#### 4.1.1. MMSE

This screening tool is effective for cognitive impairment in older adults and is achieved best when done routinely, systematically, and thoroughly [140]. Decreasing scores of the repeated test showed cognitive deficits or deterioration in cognition [141]. The validity of the MMSE as a screening test for dementia depends on the educational level [142].

#### 4.1.2. CSF Assay for Aβ

In AD, cerebral accumulation of amyloid beta is thought to be the starting process [143]. The intercellular space in the brain has always direct contact with CSF, any biochemical changes in the brain may be reflected by CSF analyses [144]. In AD patients, the cerebrospinal fluid concentration of Aβ_42_ significantly decreased [145].

#### 4.1.3. MRI for Brain Volume

MRI can detect brain abnormalities associated with mild cognitive impairment. An MRI of the brain allows a provider to assess the neurodegeneration in the early stages of the disease. In broad terms structural MRI in AD can be categorized into (a) assessing atrophy (or volumes) and (b) alteration in tissue characteristics that cause signal alterations on certain sequences [146]. In the early stages of Alzheimer’s disease, an MRI scan of the brain may be normal while in later stages, MRI may show a decrease in the size of different areas of the brain [146].

#### 4.1.4. PET Scan for Aβ Plaques and Glucose Metabolism

Positron emission tomography (PET) is a sensitive radionuclide imaging technique, which provided opportunities to detect Aβ plaques of AD [147]. Currently, a variety of chemical classes of amyloid tracers including ^11^C-PIB, ^18^F-flutemetamol, and ^11^C-AZD2184), stilbenes (^18^F-AV-1, ^18^F-AV-45, and ^11^C-SB-13), benzoxazoles (^11^C-BF-227 and ^18^F-BF-227), and benzofurans (^18^F-AZD4694) are used for PET and they have different mechanisms of action of binding to fibrillar beta-amyloid as well as other forms of Aβ [146,148]. In AD, reduced glucose metabolism due to reduced cellular activity is a major feature, and measurement of changes in glucose metabolism is allowed by ^18^F fluorodeoxyglucose (FDG) in the brain [148]. 

### 4.2. Amyloid Beta Therapeutics

**Figure 7:**
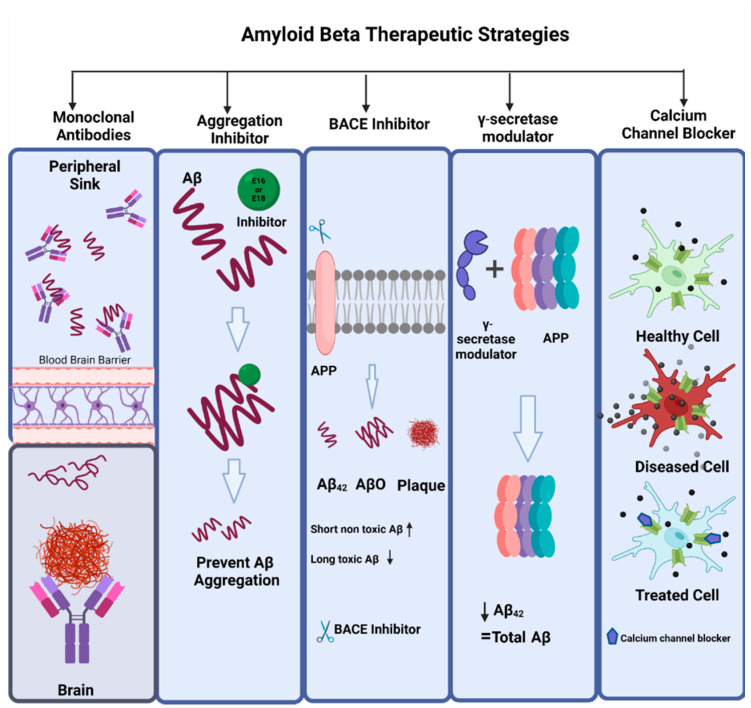
Amyloid beta therapeutic strategies. The commonly used therapeutic strategies for AD that targets amyloid beta are amyloid monoclonal antibodies, aggregation inhibitors (e.g., E16, E18), BACE inhibitors (e.g., CNP 520, MK8931, RIPK1), γ-secretase modulators, and calcium channel blockers (isradipine, nimodipine, verapamil, diltiazem).

The drugs that have been approved for the treatment of AD to date, address only clinical dementia stages of the disease and work by alleviating the behavioral symptoms and cognitive dysfunction by targeting underlying neurochemical systems. In the last two decades, advances in research have reshaped the conceptual framework of AD, and now the therapeutic aims include clinical biological constructs along with prodromal, preclinical, and dementia stages [58]. The types of compounds that work as anti-amyloid are usually amyloid monoclonal antibodies, aggregation inhibitors, BACE inhibitors, γ-secretase modulators, calcium channel blockers, antivirals, amyloid vaccines, and receptors for advanced glycation end products (RAGE) antagonists [149] (Figure 7).

The sAPPα fragment produced by α-secretase upon APP processing in the nonamyloidogenic pathway has neuroprotective properties, and β- and γ-secretases in amyloidogenic APP processing produces neurotoxic Aβ peptide that also has neuroprotective qualities and thus can be drug targets. Since in AD, the imbalance among APP processing pathways leads to an increase in Aβ production, the compounds that can either inhibit β- and γ-secretases or can activate α-secretase might be of interest for their therapeutic potential. Moreover, upregulating enzymes responsible for Aβ degradation can also be used as therapeutic candidates as they can maintain a balance between the production and degradation of Aβ peptide [150].

Alzheimer’s disease being non-recoverable is not only prevalent in the old age population and still, after decades of research, but its treatment is also limited in its efficacy. This poor efficacy is due to many biological factors including peripheral side effects, the blood–brain barrier, and drugs’ inability to reach the target brain sites [151]. The scientific efforts of the last two decades in developing a potent therapy for AD have identified the prevention of Aβ accumulation as the main target of most drugs tested. However, many other molecular targets like inflammation, tau pathology, oxidative stress, 5-HT2A receptor, and ACE inhibitors have gained attention in recent years after Aβ-based trials failed [152]. Like mitochondrial oxidative stress in neurons after Aβ accumulation can be inhibited by an influx of calcium into the mitochondria and by treatment with mitochondria-targeted antioxidant SS31 which is a synaptic peptide [153]

Neurodegenerative disease patients exhibit mitochondrial dysfunction. A small inhibitor of mitochondrial division, Mitochondrial division inhibitor-1 (Mdivi-1), plays multiple roles in mitochondria like ATP production, mitochondrial autophagy, Ca2+ homeostasis, and immune responses this molecule has AD’s therapeutic potential [154]. A recent study has revealed that hypoxia and ischemia are activators of the extracellular signal-regulated kinase (ERK) which is a feature of AD pathogenesis. Activated signal-regulated kinase phosphorylates Drp1, Dynamin-related protein 1, and promotes mitochondrial fission. Mdivi-1 inhibits ischemia and hypoxia-induced Aβ generation and mitochondrial fission [155].

Dopamine-derived structure, DDQ (diethyl (3,4-dihydroxyphenethylamino)(quinolin-4-yl)methylphosphonate), displayed the best binding energy and hinders active sites of Drp1 and Aβ in molecular docking studies for treating AD [156]. Studies conducted by our lab suggest DDQ has anti-amyloid beta and anti-aging characteristics thus making it a strong candidate in the treatment of AD by reducing amyloid-beta- and age-induced toxicity in the brain [157]. There are only a few treatments approved by Food and Drug Administration that include N-methyl D-aspartate receptor antagonists (memantine), and acetylcholinesterase inhibitors (rivastigmine, donepezil, and galantamine) [158]. However, these treatments being single-targeted only relieve symptoms and cause adverse side effects such as insomnia, headache, diarrhea, hepatotoxicity, and sickness [159].

One of the most extensively developed anti-Aβ therapeutic approaches is immunotherapy, which includes inducing passive immunity through monoclonal antibody administration [160]. The anti-Aβ monoclonal antibodies that have been recently used for the treatment of AD are bapineuzumab, aducanumab, solanezumab, gantenerumab, and lecanemab [161]. Aducanumab (Aduhelm), a monoclonal antibody, is one of the latest drugs approved by the Food and Drug Administration (FDA). Aducanumab is manufactured by Biogen and Eisai and claims to reduce amyloid accumulation in the brain [162]. However, previous studies on AD have not proved that reducing amyloid accumulation from the brain results in recovery for the patients [163]. Aducanumab selectively targets aggregated Aβ that includes Aβ plaques and Aβ oligomers [164]. Gantenerumab also targets Aβ oligomers and fibrils, crenezumab targets Aβ oligomers, and solanezumab targets Aβ monomers [165]. Lecanemab, a humanized IgG1 of the mouse, binds to Aβ protofibrils [166]. Recent randomized clinical trials of lecanemab demonstrated a reduction in brain amyloid by administering 10-mg/kg lecanemab biweekly [167]. It is necessary to conduct more clinical trials on the efficacy of lecanemab.

### 4.3. Why Amyloid Beta Therapeutics Failed

Most of the treatments for AD have mainly tried to reduce Aβ levels in the brain, some compounds achieved the target, but none has produced clinically significant results [168]. The monoclonal antibodies solanezumab, bapineuzumab, gantenerumab, and crenezumab target different Aβ isoforms and their elimination [169]. Such drugs could not succeed, and the reason could be that in the symptomatic phase Aβ may not correlate with cognitive decline. The absence of a disease-modifying treatment for AD is still a challenge to overcome. Some of the issues in drug development that affect clinical trials are as follows

The key hallmarks of AD, amyloid plaques, and aggregated tau are major components of the amyloid hypothesis and most of the drugs to date have been focused to reduce amyloid deposits in the brain but showed no success in reversing cognitive decline. We may assume doubt the validity of the amyloid hypothesis or maybe there comes a phase when AD becomes independent of amyloid beta deposits [149].

One of the reasons for failed therapeutics might be that drugs were targeting the wrong pathological substrate, for instance, in the case of monoclonal antibodies should they be targeting Aβ plaque, monomeric Aβ, or oligomeric Aβ, and what terminus of the Aβ domain should be the target [169]. The anti-amyloid antibodies might have poor brain penetration, lack of sustained and robust inhibition of soluble Aβ moieties, and patient heterogeneity also leads to failed amyloid beta therapeutics [170].

Many studies to date have reported amyloid-beta peptide as a memory enhancer. Aβ only becomes neurotoxic when it is produced in excess. This is also an important factor that leads to the failure of therapeutics. If the therapeutics aims to lower the Aβ levels in the brain, it would disrupt the physiological role of Aβ which is to maintain memory [171].

## 5. Conclusions and Future Directions

Alzheimer’s disease is one of the most common neurodegenerative diseases in elderly people, and Aβ accumulation plays a major role in the pathogenesis of this disease. A balance between the production and clearance of Aβ is needed to maintain a steady state in the cells. Since Aβ starts to appear way before the appearance of AD’s clinical symptoms, this peptide is an imported biomarker that can be used to predict disease. Therefore, research on Aβ’s biology, its role in cognitive decline, identifying its receptors, and the evolution of Aβ-based therapeutic approaches for treating AD are of paramount importance. Moreover, much of the focus of Aβ-based therapeutics has been on controlling the production and clearance of Aβ. The Aβ peptide plays many important roles like neuroprotection, memory enhancer, neural growth, and repair, and thus lowering the levels of Aβ in AD patients might not work as this strategy interferes with the normal physiological functions of Aβ in the brain. Despite extensive research on AD and the evolution of treatments, the exact neuropathological mechanism is still unclear. Animal models based on Aβ neurotoxicity have routinely been used to understand and study the development and treatment of AD. Still, more research and understanding are needed to determine the role of Aβ in AD progression and for developing an effective treatment to cure Alzheimer’s disease.

## Figures and Tables

**Table 1:** Genes linked to Aβ homeostasis.

Expression	APP, PSEN1, PSEN2, ADAM10
Transmission	APOE, CLU, SORL1
Degradation	PICALM, SORL1, CD33, BIN1, CD2AP, ABCA7, RIN3, CLU, PTK2B

## Data Availability

Not applicable.

## Abbreviations

ABCA7	ATP-binding cassette sub-family A member 7
AChRs	Neuronal-Type Nicotinic Acetylcholine Receptors
AD	Alzheimer’s Disease
ADAM10	Disintegrin and metalloproteinase domain-containing protein 10
AICD	Amyloid Precursor Protein Intracellular Domain
APOE	Apolipoprotein E
APP	Amyloid Precursor Protein
ATP	Adenosine triphosphate
Aβ	Amyloid Beta
BACE	Beta-Site Amyloid Precursor Protein Cleaving Enzyme
BIN 1	Bridging Integrator 1
CD2AP	CD2 Associated Protein
CD33	Sialic Acid Binding Ig-Like Lectin 3
CLU	Clusterin
CNS	Central Nervous System
CSF	*C* *erebrospinal fluid*
CTFα	C-terminal fragment alpha
CTFβ	C-terminal fragment beta
DDQ	diethyl (3,4-dihydroxyphenethylamino)(quinolin-4-yl)methylphosphonate
Drp1	Dynamin related protein 1
EphB2	Ephrin type-B receptor 2
ERK	Extracellular Signal-Regulated Kinase
F-Actin	Filamentous Actin
FDA	Food and Drug Administration
FDG	Fluorodeoxyglucose
IL-1β	Interleukin 1 beta
IL-6	Interleukin 6
LilrB2	Leukocyte Immunoglobulin-Like Receptor B2
Mdivi-1	Mitochondrial division inhibitor-1
MMSE	Mini-mental state examination
MRI	Magnetic resonance imaging
NgR1	Neuronal Nogo-66 receptor 1
NMDAR	N-methyl-d-aspartate receptors
PET	Positron emission tomography
p-tau	Phosphorylated Tau
PICALM	Phosphatidylinositol Binding Clathrin Assembly Protein
PirB	Murine paired immunoglobulin receptor B
PPARs	Peroxisomal Proliferators-Activated Receptors
PS1	Presenilin 1
PS2	Presenilin 1
PTK2B	Protein Tyrosine Kinase 2 Beta
RAGE	Receptor for advanced glycation endproducts
RIN3	Ras Furthermore, Rab Interactor 3
ROS	Reactive Oxygen Species
sAPPα	N-terminal fragment Amyloid Precursor Protein α
sAPPβ	N-terminal soluble Amyloid Precursor Protein β
SORL 1	Sortilin-related receptor 1
TBI	Traumatic brain injury
TNF-α	Tumor necrosis factor alpha
α	Alpha
α7-AChRs	Alpha7 Acetylcholine Receptors
β	Beta
γ	Gamma
